# Cytotoxicity Evaluation of The Bioresorbable and Titanium Plates/Screws Used in Maxillofacial Surgery on Gingival Fibroblasts and Human Mesenchymal Bone Marrow Stem Cells

**DOI:** 10.22074/cellj.2020.6409

**Published:** 2019-12-15

**Authors:** Masoud Vatani, Mohammad Hossein Beigi, Fatemeh Ejeian, Ahmad Mottaghi, Afshin Yadegari-Naeini, Mohammad Hossein Nasr-Esfahani

**Affiliations:** 1. Department of Oral and Maxillofacial Surgery, School of Dentistry, Islamic Azad University, Khorasgan Branch, Isfahan, Iran; 2. Department of Cellular Biotechnology, Cell Science Research Center, Royan Institute for Biotechnology, ACECR, Isfahan, Iran

**Keywords:** Bone Marrow Stem Cell, Cytotoxicity Test, Dental Implant Materials, Fibroblast Cells

## Abstract

**Objective:**

Bioresorbable and titanium plates/screws are considered as a standard treatment for fixation of the bone segments of craniofacial area and paying attention to their biocompatibility is an important issue along with other aspects of application. The purpose of the study was to evaluate the cell viability of two types of plate and screw used in maxillofacial surgeries in contact with gingival fibroblasts and bone marrow stem cells.

**Materials and Methods:**

In this experimental study after extraction and cultivation of cells from healthy human gingival tissue and alveolar bone of jaw, cytotoxicity of device was evaluated. In direct contact method, samples had near vicinity contact with the both cell lines and in indirect contact method, by-products released, like ions, from samples after 8 weeks were used to assess cytotoxicity. Then cytotoxicity was evaluated on the 2^nd^, 4^th^ and 6^th^ day with MTS tests and microscopy. The data were analyzed by one-way ANOVA and independent t tests.

**Results:**

There was a statistically significant difference between the German plate and screw and all the samples studied on day 6 (P<0.05). Furthermore, a statistically significant difference was observed between both metal samples and both bio-absorbable samples on day 6 and both cell lines (P<0.05). Comparisons between the two groups with each other for both cell lines on the 6^th^ day were statistically significant (P<0.05).

**Conclusion:**

Our results suggest that that cytotoxicity of biomaterial, from different brands, were not similar and some of the biomaterial showed lower degree of toxicity compared to others and specialist using these products showed be aware of this differences. Our investigation indicates more biocompatibility of bioresorbable plates and screws compared to titanium. In addition our results suggest that biomaterials were not completely neutral.

## Introduction

Primary stability and appropriate contact between fractured bone segments are essential for bone remodeling, maturity and reconstruction following maxillofacial trauma, orthognathic surgery and Healing of pathologic defects. Therefore, over centuries, clinicians have paid particular attention to these issues and developed various types of plates and screws, splints, arch bars and inter-maxillary wires in different sizes, shapes, and thicknesses. The introduction of new materials such as polymers, composites, and compound alloys during the recent decades have led to a great revolution in production and application of wide range of innovative devices in this field (1).

Different metals from stainless steel and commercial titanium alloys to nickel-chromium-cobalt and titaniumaluminum-vanadium alloys have long been used in the reconstruction of dental structures and bone tissues. Titanium alloys have been widely used in implantology for over seven decades. In maxillofacial surgery, titanium alloys are largely utilized in production of plates and screws, reconstruction meshes, and even jaw distraction devices (2). Moreover, development and progress of material engineering and clearer understanding of atomic and molecular structures of materials have resulted in production of novel biomaterials and absorbable polymers such as poly-lactic acid, poly-glycolic acid and their copolymers. Based on the unique molecular structures of these materials and their interactions with living tissues, several types of absorbable sutures and plates and screws have been produced and applied in craniofacial and orthopedic surgeries (1), specially . for the stabilization of fracture segments and osteotomy sites and internal fixation (3).

Following the development of any biomaterial, its stability, aesthetic and functional aspects, and biocompatibility should be regularly assessed by both the manufacturers and clinicians (4). Considering the improvement of international health and safety standards, governments and organizations pay utmost attention to safety of medical equipments and implantable devices. Hence, before clinical application in humans, biocompatibility of all materials is widely evaluated through standardized tests (5). In addition to technological advancements in production of instruments and biomaterials, an increasing diversity of commercial products are produced by different companies. In fact, various brands and novel products are distributed in the global market in response to the emerging global medical demands. Considering these products are in short-term or long-term contact with biological environments and their byproducts will be released after their usage and become in contact with surrounding tissue, therefore development of biocompatible materials which yield appropriate biological responses and minimize possible health risks for the patients is of paramount importance. Despite the importance of this issue, biocompatibility of some implantable devices has not been thoroughly investigated. According to available research, corrosion of implanted metallic devices and chronic exposure to their derivatives, cause acute or chronic toxicity. The consequent oxidative changes taking place in vicinity of metallic bonds of these materials can induce changes in biological molecules such as DNA and could subsequently lead to a wide range of diseases including cancers (6).

Analysis of the ions released form implanted metals indicates the potential of these ions for localized accumulation in patients’ blood, serum, or different organs (7). Researchers have long agreed on the release of titanium ions from implanted titanium alloys and monitored accumulation of these ions in patients’ lymph nodes and various organs (e.g. liver, gallbladder, and lungs), and even serum and urine (8, 9). Even the lower concentrations of metal ions can inhibit half of cellular activities and titanium and cobalt inhibit cell-specific functions including alkaline phosphatase activity, extracellular calcification, and bone-specific gene expression (10). Based on available evidence, longterm release of aluminum and vanadium from titanium alloys would cause peripheral neuropathy, osteomalacia and Alzheimer’s disease (11). Bio-absorbable implants are recognized as foreign body by the organism. Furthermore, degradation of these materials leads to release and accumulation of acidic byproducts and cause aseptic inflammation in the host’s responses, cytotoxicity, and changes in cell behaviors (12). Additionally, the complexity of healing and regeneration processes of both soft and hard tissues at surgical site also depends on the type of biomaterial used and this has become for global market. Considering these facts, this study was designed to evaluate the biocompatibility and cytotoxicity of four well-known brands applied in maxillofacial treatments through both direct and indirect contact with two cell types, including human gingival fibroblasts and human bone marrow stem cells.

## Materials and Methods

### Ethical consideration

The experiments were approved by the Ethical Committee of Isfahan Medical University (IR.MUI. REC.1395.4.040). Before surgery to obtain human gingival tissues and alveolar bone marrow stem cells patients were informed regarding the aim of the study and informed consent form was signed with each individual. Healthy human gingival tissue was obtained from 5 patients undergoing crown lengthening surgery at the Department of Oral and maxillofacial Surgery, Faculty of Dentistry, Isfahan Azad University, Iran and alveolar bone marrow stem cells were obtained from 7 patients undergoing orthognathic surgeries in Amin Hospital (Isfahan, Iran).

### Isolation and cell culture of gingival fibroblast cell

**Fig 1:**
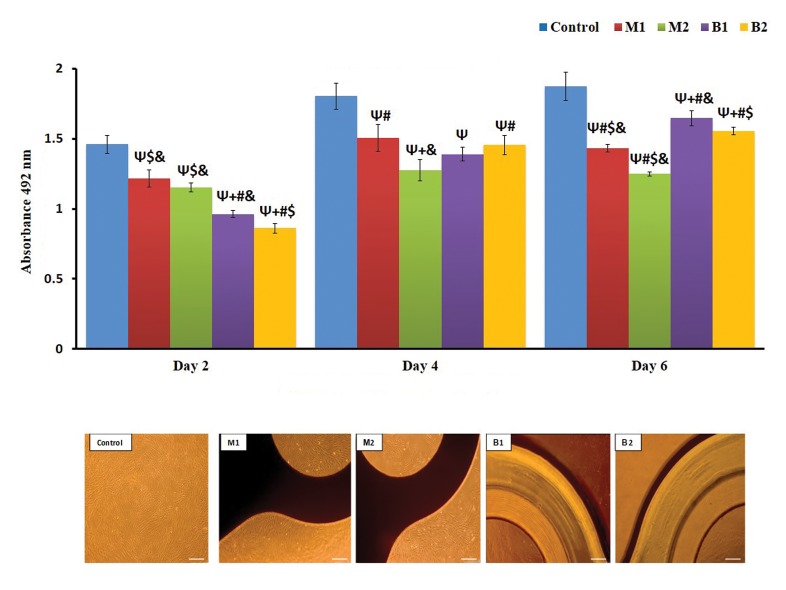
Effect of direct contact with HGFs in MTS assay and phase‐contrast microscopy images (scale bar: 200). Ψ; Indicates statistically significant difference compared with control group at P<0.05, +; Indicates statistically significant difference compared with M1 group at P<0.05, #; Indicates statistically significant difference compared with M2 group at P<0.05, $; Indicates statistically significant difference compared with B1 group at P<0.05, &; Indicates statistically significant difference compared with B2 group at P<0.05, M1; Iran, M2; Germany, B1; Finland, B2; Taiwan, and HGF; Human gingival fibroblasts.

In this experimental study, after surgery all human samples were transferred to the lab Royan Institute in phosphate- buffered saline (PBS) containing 100 U/ ml penicillin, 50 μg/ml streptomycin and 0.25 μg/ml amphotericin B. The gingival tissue were thoroughly washed and cut into small pieces (0.5×0.5 mm) and placed in high glucose Dulbecco’s modified Eagle’s medium (DMEM) containing 15% fetal bovine serum (FBS), 1% L-glutamine, 100 U/ml penicillin, 50 µg/ml streptomycin and 0.25 µg/ml amphotericin B. The culture plates were incubated at 37 ° C in a humidified atmosphere of 95% air and 5% CO_2_ and daily monitored for any infection. As the hallmark of *in vitro* fibroblast isolation, primary cell outgrowth was observed after 10 days, which were labeled as passage zero (Fig.S1) (See Supplementary Online Information at www.celljournal.org). During this period the medium was replaced twice a week. Upon confluence, cells were passaged and cells from passage 3 were used for the study. Passaging will remove other cell contaminate and help to obtain uniform gingival fibroblasts with spindle shaped morphology (Fig .1). All chemicals and reagents, unless otherwise stated, were purchased from Sigma® (St. Louis, MO). Media were purchase from Gibco (USA), unless otherwise stated.

### Isolation and cell culture of alveolar bone marrow cells

On the other hand, alveolar bone fragments obtained during orthognathic surgeries were placed over mesh covered with 4-(2-hydroxyethyl)-1- piperazineethanesulfonic acid (HEPS) medium and centrifuged at 2500 rpm to with force out alveolar bone marrow cells from the bone fragments. The cells were seeded on 25 cm^2^ flasks containing DMEM medium supplemented with 15% FBS, antibiotics (penicillin 0.1 g/L; streptomycin 0.1 g/L) at 37˚C in a humidified air atmosphere containing 5% CO_2_. Upon confluence these cells were considered as passage zero. In order to evaluate the stem cell properties of harvested cells, expression of common MSC markers (CD73, CD90, and CD105) were examined after 5 passages. In addition, the multilineage potential of cells were assesses after 3 weeks induction in specific adipo and osteogenic medium (Fig.S1) (See supplementary Online Information at www.celljournal.org).

### Direct and indirect cytotoxicity assessment

In this study, four brands of plates and screws composition were used as shown with more details in Table 1. Accordingly, M1, M2, B1 and B2 products are made in Iran, Germany, Finland and Taiwan, respectively.

Initially, all titanium plates and screws were placed in double-distilled water and then immersed in ethanol for 20 minutes and washed abundantly with double-distilled water and then sterilized at 121˚C (15 minutes). On the other way, all bio-absorbable plates and screws were sterilized using ultraviolet light. Finally, all samples were washed twice with PBS prior to use. All experiments (direct and indirect cytotoxicity assessment) were carried out according to ISO 10993-5 standardized procedures and recommendations (13).

For direct cytotoxicity, the plates and screws were placed on the surface of culture plates of 12 well dishes and the results were compared with the dish with absence of these materials. Subsequently, 3×104 cells/well were added to each well (3 well/per group) and MTS assay was carried out at 2, 4 and 6 days post exposure. According to part 4.2.3.3 of ISO 10993-5, in the indirect method, pH was adjusted after incubation period, prior to cellular treatment.

Also, indirect assay was carried out according to part 8.4 of ISO 10993-5 standardized procedures (13). Preparation of condition medium performed in sterile, chemically inert, closed containers by using aseptic techniques, in accordance with ISO 10993-12. Briefly, plates and screws were added to 15 ml tubes containing DMEM for 8 weeks (14). Subsequently, this medium was supplemented with 15% FBS, antibiotics (penicillin 0.1 g/L; streptomycin 0.1 g/L). Then, the cells were seeded at density of 3×10^4^ cells/well in 12 well plate using the medium which was exposed to plates and screws. The cells were cultured at 37˚C in a humidified air atmosphere containing 5% CO_2_. MTS assay was carried out at 2, 4 and 6 days post culture. DMEM not exposed to plates and screws was considered as control group for all the experiments (ISO 10993-5).

**Table 1:** Profile screws and plates used


	Type of materials	Form/Diameter	Ingredients	Manufacturers	Application

M1	Titanium alloy plate and screw	Plate: 4 holes straight 2 mm Screw: 7×2 mm	Ti-6Al-4V	Persian tohid medical, Iran	Craniofacial osteosynthesis
M2	Titanium alloy plate and screw	Plate: 4 holes straight 2 mm Screw: 7×2 mm	Ti-6Al-4V	Mondeal, Germany	Craniofacial osteosynthesis
B1	Plate and screw bioabsorbable	Plate: 4 holes straight 2 mm Screw: 7×2 mm	17% L-lactic acid copolymer,78.5% D,L-lactic acid copolymer,4.5% trimethylene carbonate monomers	Inion CPS, Tampere Finland	Craniofacial osteosynthesis
B2	Plate and screw bioabsorbable	Plate: 4 holes straight 2.5 mm Screw: 7×2 mm	90% L-lactide acid copolymer10% D,L lactide acid copolymer	Biotech one inc.Bonamates series, Taiwan	Craniofacial osteosynthesis


### MTS assay

MTS assay was a colorimetric assay for assessing cell metabolic activity. Micro plate Reader (Fluostar Optima, BMG Lab Technologies, Germany) at 492 nm was used to analyze the absorbance. Results were normalized as the ratio of main medium without cells and cell viability was calculated. It should be noted that on the 6th day and in both methods, the phase contrast microscopy was used to assess the quality of cells.

### Statistical methods

Statistical analysis was performed using SPSS software version 18 (IBM, USA). One-way ANOVA test was adopted to quantitatively compare among each sample and control group (more than two groups) in terms of cytotoxicity. Pairwise comparisons of the groups (Titanium alloy and bio-absorbable plates and screws) in terms of cytotoxicity were performed using independent t test. Significance was accepted at a level of P<0.05.

## Results

### Direct contact of plates and screws with human gingival fibroblast

**Fig 2:**
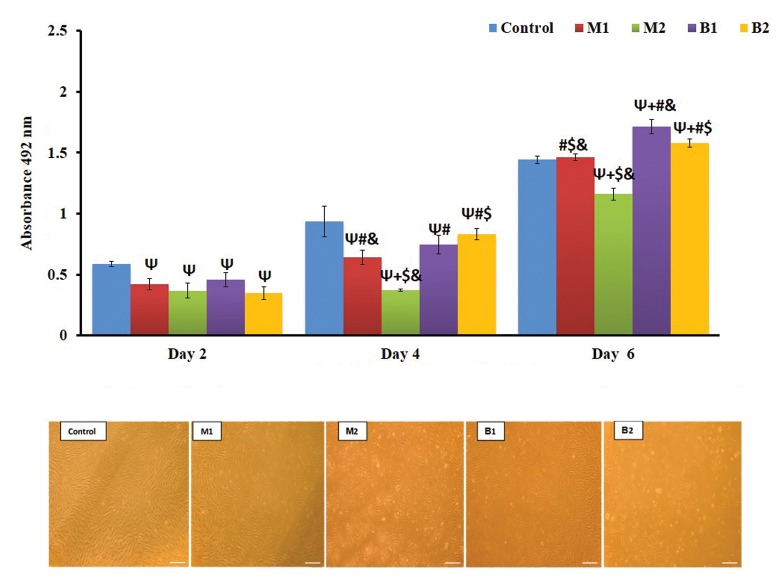
Effect of indirect contact with HGFs in MTS assay and Phase‐contrast microscopy images (scale bar: 200 μm). Ψ; Indicates statistically significant difference compared with control group at P<0.05, +; Indicates statistically significant difference compared with M1 group at P<0.05, #; Indicates statistically significant difference compared with M2 group at P<0.05, $; Indicates statistically significant difference compared with B1 group at P<0.05, &; Indicates statistically significant difference compared with B2 group at P<0.05, M1; Iran, M2; Germany, B1; Finland, B2; Taiwan, and HGF; Human gingival fibroblast.

The results showed a significant difference between the control group and all plate and screw samples (P<0.05) on the second, fourth and sixth days. All plate and screw samples revealed significant differences with each other except between M1 and M2. Moreover, independent t tests, showed a significant difference in cytotoxicity between the two groups of metallic and bio-absorbable plates and screws (P<0.05). While, the M2 sample just showed significant differences with the M1, B2 sample showed no significant differences between the bio-absorbable and metallic plates and screws in terms of cytotoxicity on fourth days of cell culture (P<0.05). Furthermore, all samples were showed significantly difference with each other (P<0.05) and no significant difference in cytotoxicity was observed between the bio-absorbable and metallic samples on the day 6 (P<0.05). Microscopic evaluation of direct contact of plates and screws with HGFs revealed that the control group contained a high density (viability) of fibroblasts cells which might be similar to B1 bio-absorbable plate and screw samples which also showed to contain cells on their surface. Unlike the control group, lowest cell density was observed in M2 group. The differences between groups and their significance, are shown in Figures 1 and 2.

### Indirect contact of plates and screws with human gingival fibroblast

Results indicated that there were significant differences between the control group and all plate and screw samples except with M1 on day six. However, no statistical differences were found in pairwise comparisons of all four plate and screw groups (P<0.05). On the fourth day, pairwise comparisons of the samples indicated that the M2 group was significantly different from all other plate and screw samples and there were no significant differences between the B1 with B2 and M1 groups. However, a significant difference was observed between M1 with B2. On the other hand, significant differences were found between all samples on the day six (P<0.05). Also, the results of independent t-test revealed that the bio-absorbable and metallic plates and screws had significant differences in terms of cytotoxicity on day four and six (P<0.05).The microscopic evaluation also confirmed the MTS test results which means that M2 group contained the lowest cell density with a higher number of dead cells than the other groups.

### Direct contact of plates and screws with alveolar bone marrow cells

**Fig 3:**
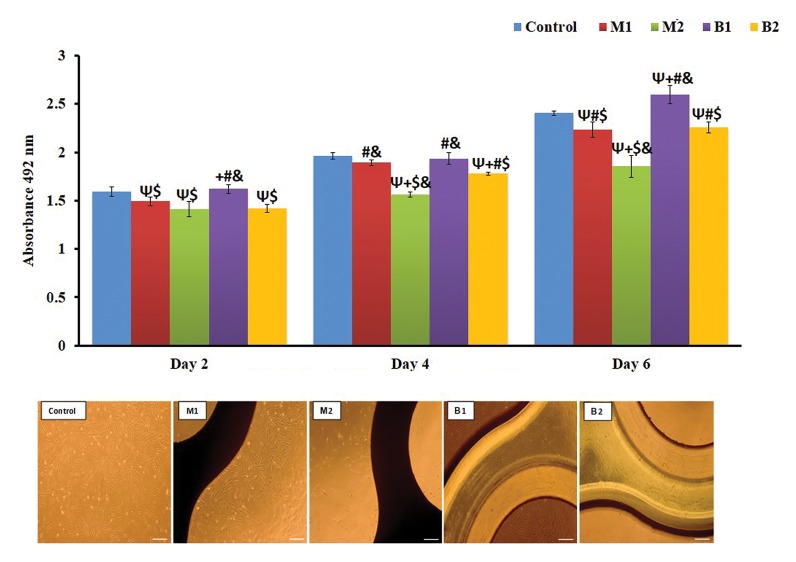
Effect of direct contact with bone marrow stem cells (BMSc) in MTS assay and Phase‐contrast microscopy images. Ψ; Indicates statistically significant difference compared with control group at P<0.05, +; Indicates statistically significant difference compared with M1 group at P<0.05, #; Indicates statistically significant difference compared with M2 group at P<0.05, $; Indicates statistically significant difference compared with B1 group at P<0.05, &; Indicates statistically significant difference compared with B2 group at P<0.05, M1; Iran, M2; Germany, B1; Finland, and B2; Taiwan.

The results of direct contact with bone marrow stem cells (BMSCs) showed a significant statistical difference between control group and all the other groups except with M1 and B1 groups on the fourth day and B1 on the second day (Fig .3). Pairwise comparisons of the samples did not show significant differences between the M2 with M1 and M2 with B2 plate and screw samples. However B1 bio-absorbable samples were significantly different from all other samples on second day (P<0.05). The results of the fourth day showed that there were no significant differences between M1 with B1 groups and in contrast, significant differences were observed between the other groups (P<0.05). The results of ANOVA, revealed that there was significant deference between the control group and all samples (P<0.05). In addition, M1 and B2 samples were almost similar (P<0.05) and other samples had significant differences with each other on sixth day (P<0.05). The results of independent t-tests showed that there were significant differences between the metallic and bio-absorbable samples the fourth and sixth days (P<0.05). Microscopic evaluation demonstrated that the density of BMSCs around and even on the B2 bioabsorbable plates and screws was higher than the other groups. In addition, the M2 and control groups were relatively similar in the density of cells and the M2 had the lowest cell density (Fig .3).

### Indirect contact of plates and screws with alveolar bone marrow stem cells

**Fig 4:**
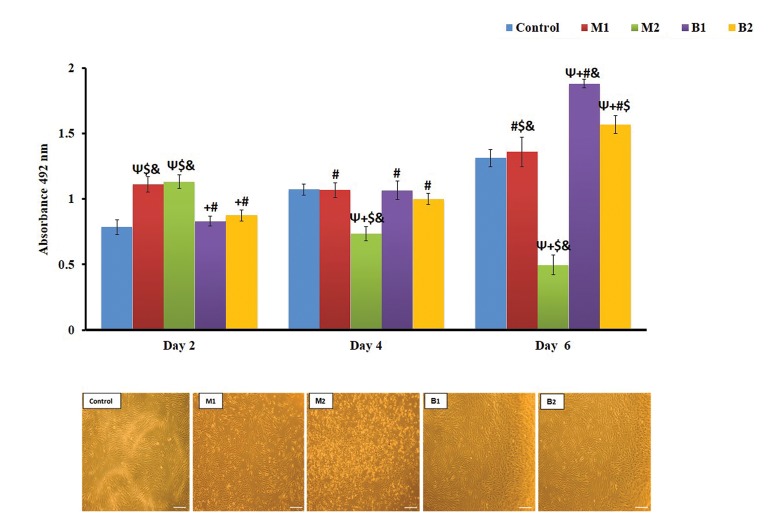
Effect of indirect contact with bone marrow stem cells (BMSc) in MTS assay and phase‐contrast microscopy images. Ψ; Indicates statistically significant difference compared with control group at P<0.05, +; Indicates statistically significant difference compared with M1 group at P<0.05, #; Indicates statistically significant difference compared with M2 group at P<0.05, $; Indicates statistically significant difference compared with B1 group at P<0.05, &; Indicates statistically significant difference compared with B2 group at P<0.05, M1; Iran, M2; Germany, B1; Finland, and B2; Taiwan.

The results indicated significant differences between the control group and all other groups, except for absorbable samples with control on second day, M1, B1 and B2 on the fourth day and M1 in sixth day (P<0.05, Fig .4). Pairwise comparisons of other samples displayed no significant differences between the M1with M2, as well as between the B1 with B2 groups (P<0.05). On the fourth day, there were no statistically significant differences between the M1, B1 and B2 with each other, but the M2 group was significantly different from all other samples (P<0.05).

Likewise, on day 4, the results showed significant differences between the M1, M2, B1, and B2 groups (P<0.05). Independent t test revealed a significant difference between the metallic and bio-absorbable groups for the all days (P<0.05). The microscopic evaluation also confirmed the MTS test results (Fig .4).

## Discussion

Nowadays, the use of bio-absorbable and titanium plates and screws in various types and forms are proposed as a gold standard to integrate and stabilize a fracture or osteotomy sites. In addition, rigid internal fixation plays a crucial role in management of reconstruction of traumatic injuries, rehabilitation of pathological defects and congenital anomalies in craniofacial area and orthognathic surgeries.

As with increased advancements in technology and biomaterials development in terms of reconstruction, replacement or repair of tissue functions in living systems, manufacturing companies and clinicians are required to consider and evaluate biocompatibility as a functional ability of a material under special conditions in the presence of an appropriate host response, in addition to considering the strength, abrasion and corrosion resistance, beauty and other practical aspects (4). Despite many studies on physical and mechanical properties and features of absorbable and titanium plates and screws used in maxillofacial region, less attention is paid to biocompatibility of these devices.

In this study, cytotoxicity of two kinds of plate and screw made of titanium alloys (Ti-6A1-4V) and bioabsorbable polymers with main structure of Poly (L-lactic acid) and Poly (D, L Lactic acid) were evaluated. Ideally, if possible, cytotoxicity tests should be selected by similar cell and tissue samples with maximum efforts to stimulate implanting and using inside of the body. The use of cell culture media is regarded as an important part of tests recommended for evaluation of biologic behaviors of materials in contact with human tissues; and primary cells have high priority in comparison with prepared cell banks in order to obtain real results and evaluate biologic behaviors and features (15, 16). So, in this study, because of close vicinity of plates and screws to bone tissues and covering mucosa, bone marrow stem cells of alveolar jaw and oral gingival fibroblast cells of human were used.

It is noteworthy that in this study, culture media with these two cell lines have been used as control group to compare cytotoxicity of plate and screw samples. Here, toxicity of plates and screws were evaluated using direct and indirect contact methods. Direct contact method has high sensitivity, and observed changes regarding cell density and morphology are representative of material’s special features during a short interval in close contact with cells. In indirect method, the effects of byproducts and materials released from samples on cells are investigated in terms of quantity and even morphology during a similar period with clinical application conditions in the body. In this study, the materials released from plate and screw samples were placed in contact with cells after an interval about 8 weeks and similar to required conditions and time in order to heal and integrate in bone segments (14).

On the other hand, MTS laboratory test was used for quantitative evaluating the survival cells in vicinity of plate and screw samples or by-products and for reducing possible errors caused by qualitative evaluation methods.

The results of direct contact of plate and screw samples with titanium alloy in two cell lines of gingival fibroblast cells and bone marrow stem cells of jaw showed a significant statistical difference compared to control group except for M1 and B1 groups with control on day 4 and B1 on day 2 in BMCs. Overall reduction in cell number compared to control group is expected as there is less area for these cells to attach unless the cells can attached to the plates and screws. This proposition is in line with cell attachment observed on B group. However, to prove that this observation or reduction in cell proliferation is not due to cellular toxicity but rather than the reduced area, the indirect culture was carried out. The results indicate that cellular proliferation was even higher or at least similar for the explants except for M2 which appear to be cytotoxic at both MTS and microscopic level in indirect method, the reduced cellular proliferation appears to be more pronounce on early days of culture (0 to day 2) compared to day 4 to 6 in HGFs a detailed explanation of which is given below.

Comparison of MTS assay between B1 and B2 with M1 and M2 in indirect method on day 6 revealed significant increase in cell proliferation in the former group (B1 and B2). Partially the same pattern existed for the direct method. Increased attachment area may also account for this observation in B compared to M group. This is in line with reports in literature that cells cannot attach to metal surfaces like titanium (17). The second reason for reduced cell proliferation in M groups is stated in the section below.

Corrosion, ionization, and abrasion of alloy samples, existence of proteins, amino acids, low concentration of insoluble oxygen, ambient temperature changes, and even higher concentration of chloride ion play an important role in ion release in adjacent tissues (18). These ionic compounds in biologic environments and plasma proteins lead to induction of thermodynamic forces for oxidationreduction reactions (19). The pH changes during the first two weeks after surgery which causes surface changes of alloys, ion release and by-products (18). Galeotti et al. (20) investigated the pH effects on biocompatibility of orthodontic mini screws in keratinocyte, human osteosarcoma, and human gingival fibroblast cultures. They found that all mini screws had tissue compatibility at pH=7 and cytotoxicity responses appeared clearly after reduced pH. Therefore, to prevent the effect of pH changes in the media which can affect cell survival and proliferation, the pH of culture medium was adjusted before exposure to cells. However, it is important to note that after contact to culture media with metal plates and screws gradual release of metallic compounds and metal ions present in these alloys and this may account for cytotoxicity observed in M group, especially M2 group. The difference between M1 and M2 is related to differences between the compositions of these two alloys, especially for vanadium. M2 probably contain a higher degree of vanadium in composition. Nevertheless the reason for higher composition of vanadium is that it improves the strength and hardness of titanium alloys to counteract the deformation of plates and screws against biting force and muscle tension on both sides of the fracture line (21). It is also important to note that vanadium is an essential micro element and plays an important physiological and pharmaceutical properties, such as insulin-like effects (22, 23). However, vanadium released faster than aforementioned alloys and at doses higher than physiological level is considered to have a high toxicity effect in comparison with other essential elements and also titanium, aluminum, nickel and cobalt (7, 18, 22, 24). On the other hand, cells with different origin are characterized by specific and sometimes different inherent features and responses in dealing with ionic metal compounds or other foreign body, and therefore the results of a cell line may not be fully consistent or comparable to another cell line, this is the reason that we used primary cell lines obtained from maxillofacial region (15, 16). In this study, based on absorbance difference one might conclude that more cellular changes were observed in bone marrow stem cells as compared to gingival fibroblast cells, which may be due to different behaviors and responses of different cell lines in direct proximity to the study materials. Nevertheless this conclusion should be taken with caution, as direct comparison between two cell lines are not possible unless cellular doubling time should also be taken into account (25) but if overlook this assumption, pre-osteoblast appear to be more sensitive than fibroblast.

The data from both direct contact and indirect methods revealed significant difference between B1 and B2 for both cell lines. The rate of proliferation was slower in B2. This is likely due to the higher biodegradation rate of B2 compared to B1 which resulted in higher rate of hydrolysis and further release of the by-products and changes in pH of the environment. These results and propositions are in agreement with microscopic observation of two cell line between the two groups.

As stated above, pH in vicinity of implanted devices may have a profound effect on cellular behavior. It is important to note that the pH in the medium, may be slightly different from the pH on surface of the implanted devices as the concentration of by-products release and therefore changes are higher in the vicinity of these medical devises (10, 26, 27). To counteract the pH shift near these medical implants, some companies have included tri-methylene carbonate in their chemical composition. According, Wake et al. (10), showed that this compound in the polymer structure has a strong buffering capacity and can neutralize the acidity shift and may protect cells from this side effect. In addition they reported that presence of inflammatory cells in the vicinity of polymers containing tri-methylene carbonate was lower than that of poly l lactic acid (PLLA) polymers. In this regard other carbonates like calcium alkaline carbonate, sodium bicarbonate and calcium bicarbonate has been added to polymers to improve buffering capacity (28).

One of the shortcoming of *in vitro* studies is that a healthy immune system along with a blood circulatory system and a healthy lymphatic drainage in the human body or every living creature is missing in this system and our study is no exemption from this shortcoming. However, it might be beneficial to investigate the effects of these plates and screws in future animal models. In this regard, selecting the most appropriate *in vivo* model is essential during the biomaterials development process to enable accurate modelling of therapeutic efficacy.

## Conclusion

Cytotoxicity testing is a mandatory part of devices in contact with living tissues. Considering the important role played by titanium and absorbable plates and screws in medicine and dentistry, especially in craniofacial surgery, therefore, it should be important for the specialist to have an insight on differential toxicity of any type of medical implant available in the market. Our results revealed different toxicity levels between different products with two primary cell lines derived from oral and maxillofacial region. Therefore, our recommendation to specialist is working with common products in their field to periodically check their cytotoxicity in order to improve the health care of their patients.

## Supplementary PDF


