# Supplementary material for: Cytotoxicity Evaluation of The Bioresorbable and Titanium Plates/Screws Used in Maxillofacial Surgery on Gingival Fibroblasts and Human Mesenchymal Bone Marrow Stem Cells
Source: Cell J. 2019 Dec 15;22(3):310–8. doi: 10.22074/cellj.2020.6409 (PMC6947002; doi:10.22074/cellj.2020.6409)
Supplement: Supplementary file 1 [file Cell-J-22-310-s01.pdf]

CELL JOURNAL (Yakhteh)

Supplementary Information for

Cytotoxicity Evaluation of The Bioresorbable and Titanium Plates/Screws Used in Maxillofacial Surgery on Gingival Fibroblasts and Human Mesenchymal Bone Marrow Stem Cells

Masoud Vatani, Ph.D.[1], Mohammad Hossein Beigi, M.Sc.[2], Fatemeh Ejeian, M.Sc.[2], Ahmad Mottaghi, Ph.D.[1]*, Afshin Yadegari-Naeini, Ph.D.[1], Mohammad Hossein Nasr-Esfahani, Ph.D.[2]*

1. Department of Oral and Maxillofacial Surgery, School of Dentistry, Islamic Azad University, Khorasgan Branch, Isfahan, Iran
2. Department of Cellular Biotechnology, Cell Science Research Center, Royan Institute for Biotechnology, ACECR, Isfahan, Iran

**Corresponding Addresses: P.O.Box: 81595158, Department of Oral and Maxillofacial Surgery, School of Dentistry, Islamic Azad University, Khorasgan Branch, Isfahan, Iran*
P.O.Box: 8159358686, Department of Cellular Biotechnology, Cell Science Research Center, Royan Institute for Biotechnology, ACECR, Isfahan, Iran
Emails: Dr.motaghi@yahoo.com, mh.nasr-esfahani@royaninstitute.org

Characterization of alveolar bone marrow stem cells

Regarding the minimal criteria of mesenchymal stem cells approved by International Society for Cell & Gene Therapy (ISCT), harvested cells were examined for expression of common mesenchymal stem cells (MSCs) markers (1). In brief, 5×10 5 /ml single cell suspensions were fixed with 4% paraformaldehyde, and so stained with particular primary and secondary antibodies against CD73, CD90, CD105, and CD45 surface markers. Flowcytometric analysis have been carried out to evaluate purity of MSCs among cultured cells after 5 passages.

On the other way, cells were treated for 3-weeks under specific adipo- and osteo-induction condition to assess their multilineage differentiation potential. Adipogenesis was induced by basal medium supplemented with 100 nM dexamethasone, 50 µg/ml β-glycerophosphate, and 50 µg/ml indomethacin, and finally assessed with Oil-Red staining of lipid droplets. Furthermore, osteogenesis was promoted by growth medium composed of 50 mg/ml ascorbic acid, 10 mM β-glycerophosphate, and 10 nm dexamethasone and differentiation efficiency was examined by Alizarin-Red staining of extracellular calcifications.

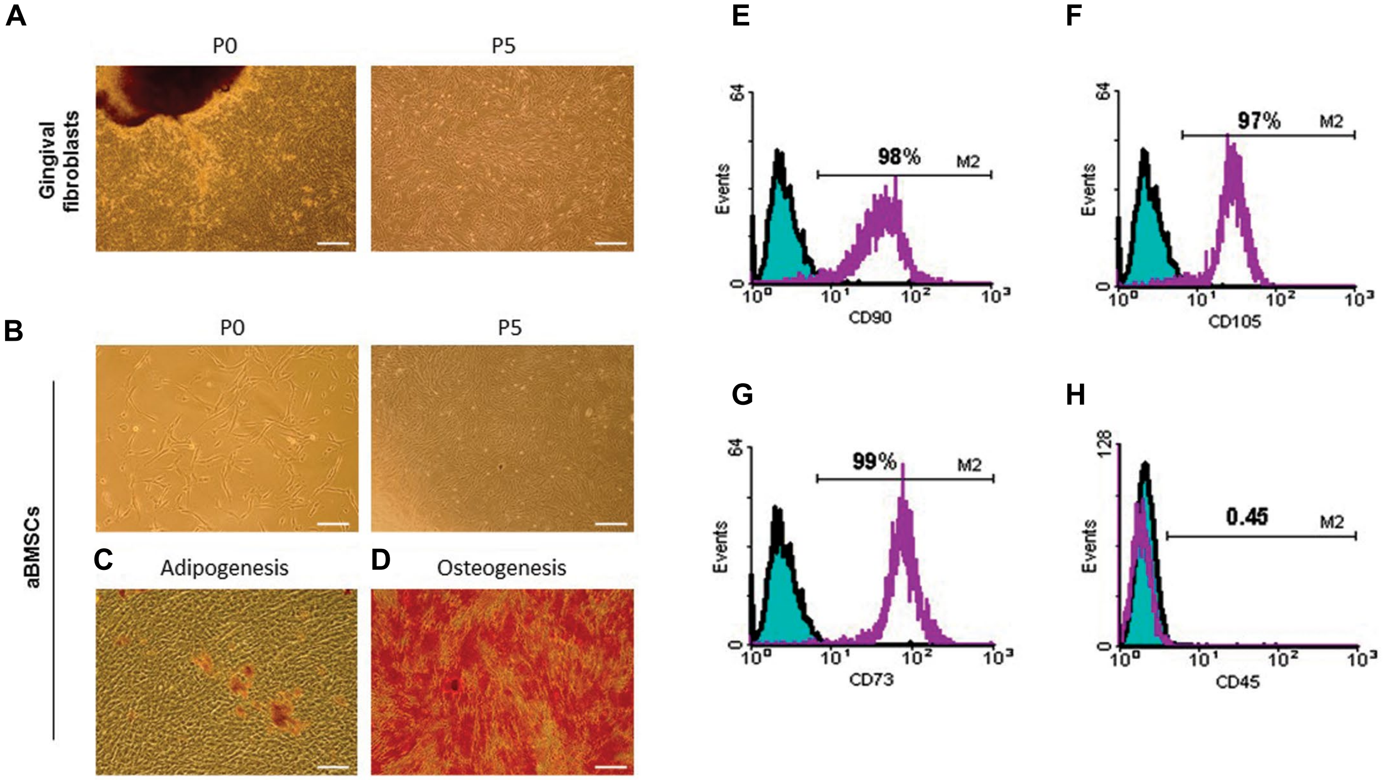

Fig.S1. Characterization of isolated gingival fibroblasts and aBMSCs. **A.** Primary cell outgrowth of was monitored from gingival explants with dominant fibroblastic morphological features after 5 passages, **B.** Extracted bone marrow stem cells show spindle-shape morphology in primary culture (p0), as well as passage 5. Following adipo- and osteogenic induction cells **C.** Lipid droplets and **D.** Extracellular calcification were respectively stained with Oil-red and Alizarin red, and **E-H.** Isolated aBMSCs revealed the extraordinary expression of CD73, CD90, and CD105, while they are clearly negative for CD45 marker.

Reference

1. Dominici M, Le Blanc K, Mueller I, Slaper-Cortenbach I, Marini F, Krause D, et al. Minimal criteria for defining multipotent mesenchymal stromal cells. The International Society for Cellular Therapy position statement. Cytotherapy. 2006; 8(4): 315-317.
